# Cognitive remediation following electroconvulsive therapy in patients with treatment resistant depression: randomized controlled trail of an intervention for relapse prevention – study protocol

**DOI:** 10.1186/s12888-020-02856-x

**Published:** 2020-09-16

**Authors:** Nele Van de Velde, Mitchel Kappen, Ernst H. W. Koster, Kristof Hoorelbeke, Hannelore Tandt, Pieter Verslype, Chris Baeken, Rudi De Raedt, Gilbert Lemmens, Marie-Anne Vanderhasselt

**Affiliations:** 1 Department of Psychiatry, Ghent University Hospital, C. Heymanslaan 10, 9000 Ghent, Belgium; 2 Department of Experimental Clinical and Health Psychology, Ghent University, Ghent, Belgium; 3 Ghent Experimental Psychiatry (GHEP) lab, Ghent University, Ghent, Belgium; 4 Department of Anesthesiology, Ghent University Hospital, Ghent, Belgium; 5 Department of Psychiatry, University Hospital (UZBrussel), Brussels, Belgium; 6 Department of Electrical Engineering, Eindhoven University of Technology, Eindhoven, the Netherlands

**Keywords:** Electroconvulsive therapy, Cognitive remediation, Major depression

## Abstract

**Background:**

Major depressive episode (MDE) is worldwide one of the most prevalent and disabling mental health conditions. In cases of persistent non-response to treatment, electroconvulsive therapy (ECT) is a safe and effective treatment strategy with high response rates. Unfortunately, longitudinal data show low sustained response rates with 6-month relapse rates as high as 50% using existing relapse prevention strategies. Cognitive side effects of ECT, even though transient, might trigger mechanisms that increase relapse in patients who initially responded to ECT. Among these side effects, reduced cognitive control is an important neurobiological driven vulnerability factor for depression. As such, cognitive control training (CCT) holds promise as a non-pharmacological strategy to improve long-term effects of ECT (i.e., increase remission, and reduce depression relapse).

**Method/design:**

Eighty-eight patients aged between 18 and 70 years with MDE who start CCT will be included in this randomized controlled trial (RCT). Following (partial) response to ECT treatment (at least a 25% reduction of clinical symptoms), patients will be randomly assigned to a computer based CCT or active placebo control. A first aim of this RCT is to assess the effects of CCT compared to an active placebo condition on depression symptomatology, cognitive complaints, and quality of life. Secondly, we will monitor patients every 2 weeks for a period of 6 months following CCT/active placebo, allowing the detection of potential relapse of depression. Thirdly, we will assess patient evaluation of the addition of cognitive remediation to ECT using qualitative interview methods (satisfaction, acceptability and appropriateness). Finally, in order to further advance our understanding of the mechanisms underlying effects of CCT, exploratory analyses will be conducted using video footage collected during the CCT/active control phase of the study.

**Discussion:**

Cognitive remediation will be performed following response to ECT, and an extensive follow-up period will be employed. Positive findings would not only benefit patients by decreasing relapse, but also by increasing acceptability of ECT, reducing the burden of cognitive side-effects.

**Trial registration:**

The study is registered with ClinicalTrials.gov. Study ID: NCT04383509

Trial registration date: 12.05.2020.

## Background

Virtually all national and international guidelines consider electroconvulsive therapy (ECT) a valuable, effective, safe and tolerable therapeutic option in cases of treatment resistant major depressive episode (MDE) and for severe and life-threatening depressive conditions (i.e., psychotic depression, high suicide risk, life threatening malnourishment) [1, 2]. During the ECT procedure, performed under general anesthesia and a muscle relaxant to minimize side effects, epileptic activity is induced/generated by a controlled passage of electric current through the brain. Even though the exact neurobiological mechanism of action remains largely unclear, antidepressant effects are attributed to changes in neurotransmitter levels, improved neuroplasticity, increased functional connectivity, and an increase in plasmatic brain-derived neurotrophic factor (BDNF). The therapeutic efficacy of ECT for MDE is characterized by a fast onset of action and high initial response rates. Meta-analyses support superiority over pharmacotherapy [3] and response rates of 74,2% for MDE in unipolar patients and 77,1% for MDE in bipolar patients are reported [4]. The pooled remission rates, even in cases of treatment resistance, are 52,3% for unipolar and bipolar patients [4].

Unfortunately, longitudinal data show low sustained response rates with relapse rates as high as 84% within the first 6 months after ECT [5]. Relapse prevention strategies are therefore key to maintain remission after successful ECT and identifying new therapeutic options are necessary. Several strategies have been put forward to prevent relapse after ECT. These include the continuation of pharmacotherapy and especially, combining an antidepressant (nortriptyline or venlafaxine) and lithium. In a 2001 study, Sackeim and colleagues reported relapse rates of 39% for the combination of lithium and nortriptyline compared to 60% for nortriptyline monotherapy over a six-month period [5]. The latter benefits have been confirmed in more recent RCTs [6, 7], and a meta-analysis found 6-months relapse rates in modern post-DSM-III studies of 37.7% for patients treated with pharmacotherapy [8]. However, there is also growing evidence on the utility of more recently developed antidepressants such as venlafaxine (225 mg/day) as they have a more favorable side-effect profile [6]. Continuation ECT (c-ECT), where patients receive continued ECT at a reduced schedule after a successful index ECT course, is another valuable relapse prevention strategy. Relapse rates, 37.2% over a 6-month period, are comparable to continuation pharmacotherapy [8]. Kellner et al. (2006) reported even more favorable outcomes by combining continuation pharmacotherapy (venlafaxine + lithium) with ECT tapering and c-ECT as needed in a geriatric population [6].

Yet, given that current 6-month relapse rates remain high at 37,7% [8] despite the use of existing relapse prevention strategies, there is an urgent need for novel interventions. Hereby, interventions, based on the remediation of cognitive deficits that occur during and following ECT treatment, may offer an interesting opportunity. Cognitive impairment is an important symptom cluster in affective disorders and may continue to affect functional outcomes in a negative matter even after achieving remission of mood symptoms [9]. Bortolato and colleagues introduced the concept of cognitive remission as an additional aim in the treatment of affective disorders [10]. Importantly, cognitive side-effects after ECT are perceived by patients as the most disturbing due to their negative impact on quality of life [11] and a descriptive systematic review by Rose and colleagues showed subjective memory loss in 29 to 55% of patients 6 months after ECT [12]. In fact, the reluctance patients express to undergo a treatment with ECT is partially based on the expected cognitive side effects.

In an extensive meta-analysis that included 84 within-subjects designed studies (a total number of almost 3000 subjects and 24 different cognitive variables) using optimal stimulation parameters, Semkovska & McLoughlin found a short-term decrease in a broad range of cognitive control processes [13]. Cognitive control refers to processes that allow information processing and behavior to vary adaptively and flexibly depending on current goals [14], and includes a broad class of mental operations such as (working) memory, attention, and concentration. These cognitive deficits remain within 3 days following ECT treatment, followed by recovery in the two subsequent weeks except from deficits in episodic retrograde (i.e., autobiographical) memory. Most importantly, these cognitive side effects of ECT, even though transient, might trigger mechanisms that increase relapse in depressed patients. Indeed, based on the available cross-sectional, prospective and longitudinal research, reduced cognitive control is an important neurobiologically driven vulnerability factor for depression (for a review, see De Raedt & Koster, 2010) [15]. Intensive research has shown that deficient cognitive control is a crucial vulnerability factor for recurrent depressive episodes. Moreover, repetitive negative thinking (RNT) is a key vulnerability mechanism in depression and research suggests that the inability to interrupt RNT is linked to inadequate cognitive control [16–19].

Recently, researchers have started to investigate computerized cognitive control training to improve depression and cognitive risk factors with encouraging results. Cognitive Control Training (CCT) uses a basic cognitive task that strongly loads on working memory and cognitive control processes, namely the adaptive Paced Auditory Serial Addition Task (aPASAT). During this task individuals are presented with a digit every 3 s and are asked to add every two consecutive digits. Task difficulty is modified based on the individuals’ current task performance, allowing training of cognitive control. This task is well-validated as a tool to remediate depression-related cognitive impairments. Moreover, the training puts low emphasis on the visual and auditory abilities of patients. Siegle et al. (2007) combined treatment as usual with several sessions of cognitive control training over a two-week period in depressed patients [20]. This approach resulted in reduced rumination and depressive symptomatology compared to a treatment as usual control group. In a later research report, Siegle et al. (2014) provided first evidence for long-term benefits of cognitive control training, showing reduced need for clinical care during a one-year follow-up period [21]. Meanwhile, other researchers have replicated beneficial effects of cognitive control training on depressive symptomatology in clinical samples [22, 23]. Positive effects were also found on stress reactivity and maladaptive emotion regulation in vulnerable people 1 month after the intervention [24]. A recent RCT in remitted depressed patients using the same training tool as we propose in the current project, showed reduced cognitive vulnerability, less residual depressive symptoms, reduced constraints in daily life and even increased resilience (measured 3 months after the intervention) [25]. Indeed, a recent systematic review (including 34 experimental studies) demonstrated that cognitive control training could be used as an add-on preventive and/or curative intervention in the treatment of depression [25]. Therefore, this cognitive remediation intervention seems to be an important non-pharmacological intervention for relapse prevention and could be promising in patients that partially or fully responded to ECT.

Provided that early studies suggest that task engagement is important for beneficial effects of CCT and facial features are an indicator of learning and engagement [26], depressive symptomatology [27, 28] and cognitive functioning [29], the current research will use facial video footage to record physiological responses during CCT. Monitoring facial expressions via videotape is a non-intrusive manner to further explore the mechanisms underlying effects of CCT. Over the years, many researchers have attempted to identify facial expressions indicative of depressive symptomology [28, 30] as well as frustration and engagement [26]. Whereas the labelling of patients’ facial expression used to be lengthy and costly operations, recent developments in technology enable us to use automated classification algorithms to gain insight in patients’ videotaped facial expressions [31]. Due to the disease burden of this specific patient group, collecting data in a passive and non-intrusive fashion (no extra constraints on patients’ workload) could bring forth novel insights in the effects of CCT and depression. In order to explore mechanism of action of this cognitive control training, exploratory analysis will be conducted with regards to video footage collected during the CCT and the active placebo phase of the study.

## Methods

### Aims

A first aim of this randomized-controlled trial is to assess the effects of cognitive control training (CCT) compared to an active placebo condition following (partial) clinical response (at least a 25% reduction of clinical symptoms) to ECT on depression symptomatology, cognition, cognitive complaints and quality of life. Secondly, given that the application of ECT is reserved for the most severely ill patient population characterized by high chronicity and that cognitive side effects due to ECT might be mechanisms that increase risk of relapse, cognitive remediation interventions targeting cognitive control mechanisms could be a promising relapse prevention strategy in patients that partially or fully responded to ECT. As such, given that the risk of relapse is greatest within the first 6 months following ECT [8], we will monitor patients every 2 weeks within that timeframe, allowing detection of potential relapse of depression. Thirdly, we will assess patient evaluation of the addition of cognitive control training to ECT using qualitative interview methods. Finally, in order to further advance our understanding of the mechanisms underlying effects of CCT, exploratory analyses will be conducted using video footage collected during the CCT/active control phase of the study.

### Hypotheses

On our primary outcome measure, depressive symptomatology as measured by HAM-D, BDI-II and RDQ, we expect a reduction in patients that received 2 weeks of CCT as compared to the active placebo condition following ECT. On our secondary outcome measures we expect [1] reduced cognitive complaints (SSMQ), [2] improved cognitive functioning (CANTAB, PASAT), [3] reduced rumination (RRS-10, 4) enhanced quality of life (QLDS) in patients that received the cognitive control training compared to active placebo. These effects are expected immediately after the CCT as well as within the 6 months follow up period. Beneficial effects will also be reflected in reduced risk for relapse of depression during the period of 6 months follow-up. Moreover, we expect more positive treatment evaluations by both patients and their peers, increased patient satisfaction and acceptability of ECT in patients that received CCT as compared to an active placebo condition following clinical response ECT.

### Study design and setting

This study uses a single blind mixed methods randomized controlled superiority trial with an embedded qualitative component. Participants will be recruited from one study site in a rural ECT-clinic in Belgium. Eligible participants will be encouraged to participate through flyers and posters in the ECT-clinic. Referring physicians will be informed of the study protocol. Participants will give informed consent for the study, cognitive control training or placebo post-ECT, prior to the start of electroconvulsive treatment.

The study has one intervention arm (cognitive control training) and one active placebo arm. All patients who give consent for participation and who fulfil the inclusion criteria will be randomized. Upon registration in the computer-based training by the allocated researcher, the computer will randomly allocate participants to either cognitive control training or placebo condition (e.a., computerized full random number generator). The researcher will monitor adherence to placebo or cognitive control training. Outcome assessors or ECT-physicians will only be involved in recruitment, ECT and post-intervention follow-up. They will not be involved during the two-week intervention phase of the study. Thus, randomization will be conducted without any influence of the principal investigators or outcome raters and both outcome assessors, ECT-physicians and participants will be blinded. Study data will be collected using digital and paper forms. Only outcome assessors and researchers will have access to the data. Data will be stored on a secure, password-controlled, server hosted at Ghent University. Qualitative interviews will be conducted in both arms after completion of CCT or placebo training. This study is in accordance with the Declaration of Helsinki (2013) and approved by the Ethical Committee of the Ghent University Hospital.

### Inclusion and exclusion criteria

Patients will be asked to participate in the current study as soon as they consent to ECT and before ECT is started. Depression diagnosis will be confirmed based on the Diagnostic and Statistical Manual (DSM-5) diagnostic criteria for major depressive episode using the screening items and depressive episode module (A) of the semi-structured Mini International Neuropsychiatric Interview (MINI v7.0.0). Both patients suffering from unipolar depression and bipolar depression are eligible. Treatment resistance, defined as non-response to two treatment trials with drugs from a different pharmacological class, each used in an adequate dose for an adequate time period in the current episode [32, 33], will be checked by the investigator (a certified psychiatrist) at inclusion. Severity of depressive symptoms will be measured by the BDI-II and the HAM-D. Patients with neurological and/or neurodegenerative disorders will be excluded using the Montreal Cognitive Assessment (MOCA< 18) at the beginning of the ECT course.

Inclusion criteria are (a) current major depressive episode with treatment resistance, (b) eligibility and consent for ECT treatment, (c) ability to consent to the study, (d) aged between 18 and 70 years old. Exclusion criteria are (a) neurodegenerative disorder or MOCA < 18, (b) catatonia, (c) schizophrenia, (d) alcohol use disorder in the previous year, (e) prior treatment with ECT, (f) insufficient computer knowledge or analphabetism.

### ECT protocol

Patients will receive ECT treatment with a MECTA SpECTrum 5000Q device based on the current standards [34, 35]: ultra-brief pulse (0.3 pulse width) with a right unilateral stimulus (RUL), a dose titrated relative to the patient’s own seizure threshold and an optimized anesthesia technique. BDI-II will be administered weekly to monitor clinical progress. If patients fail to respond after 6 RUL ECT sessions, switch to bitemporal electrode position will be considered. If there is no clinical response, ECT will be stopped after 12 sessions. If there is clinical response, ECT will be stopped when clinical remission is achieved or if there is no more clinical improvement after two subsequent sessions.

### Procedure

**Fig. 1:**
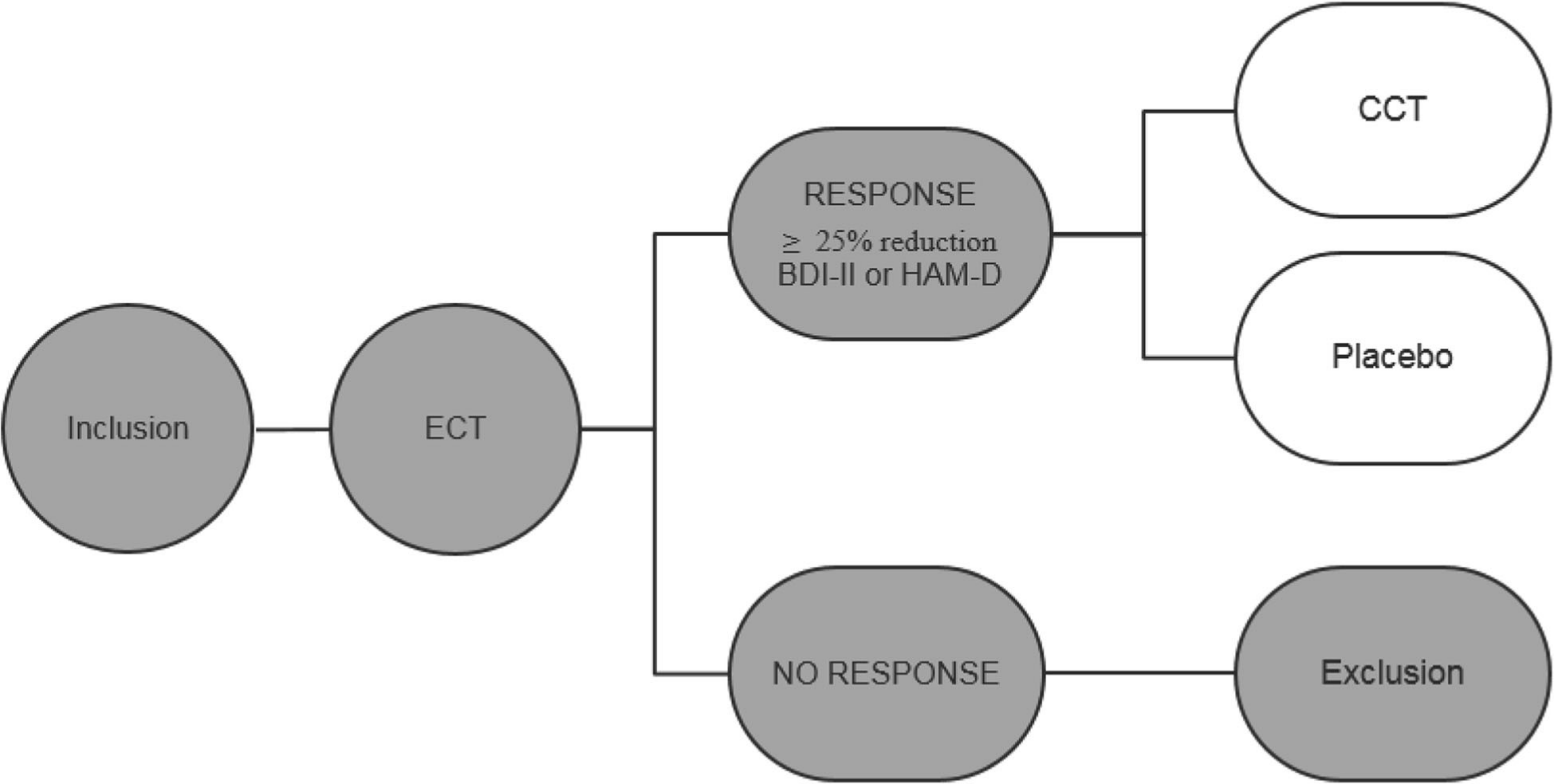
Only patients who achieve a 25% or more reduction in BDI-II or HAM-D scores [36] will be randomly assigned to one of two conditions (CCT or active control)

**Fig. 2:**
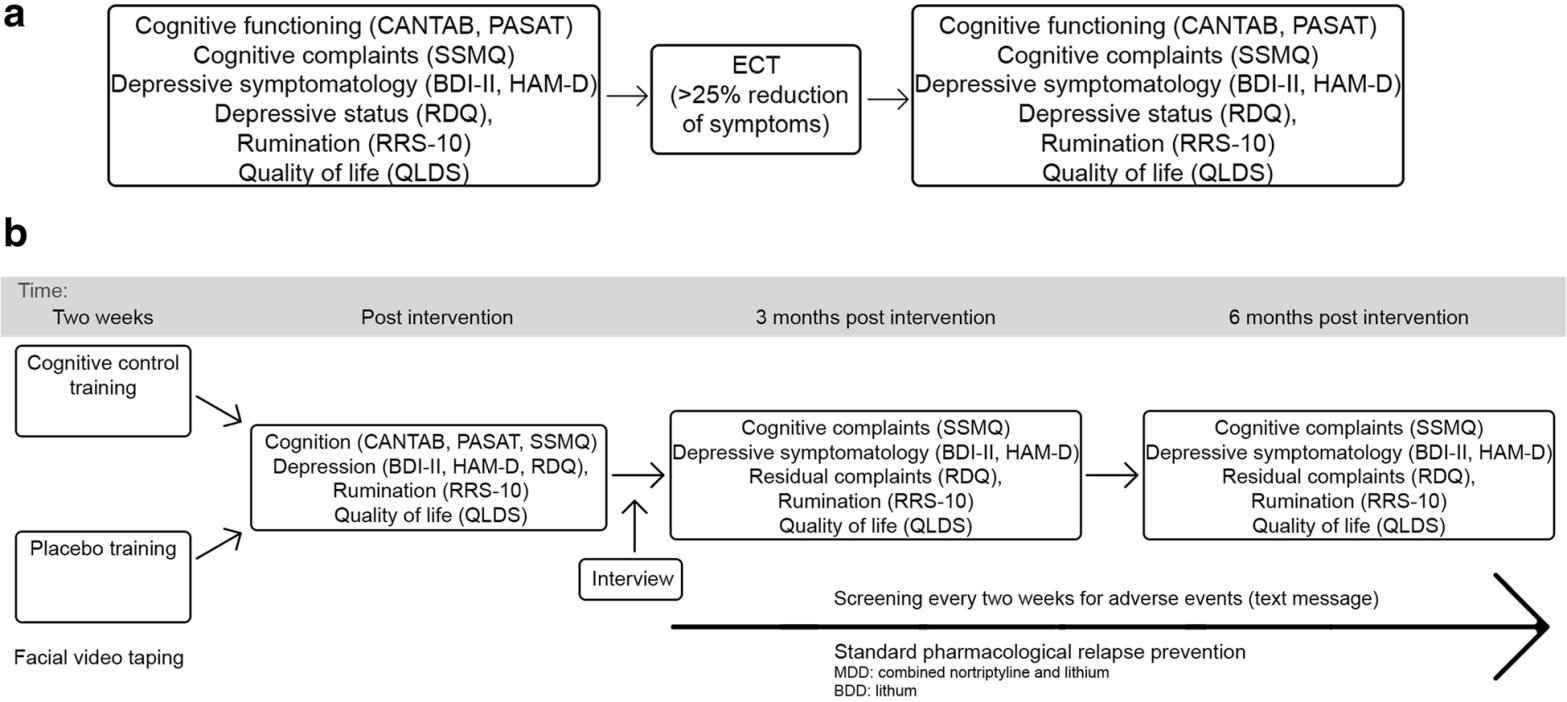
**a.** Study flow before randomized condition assignment. **b**. Study flow after randomized condition assignment

Outcome assessors and/or ECT-physicians will provide adequate verbal and written information regarding the study to all eligible patients at the ECT-clinic. Researchers will obtain written informed consent from all patients willing to participate and able to give consent. After consenting to participate and before the start of ECT treatment, all patients will complete baseline cognitive function assessment (CANTAB, PASAT), cognitive complaints (SSMQ), depressive symptomatology (BDI-II, HAM-D), depressive status (RDQ), rumination (RRS-10) and quality of life (QLDS). After ECT completion, cognitive functioning (CANTAB, PASAT), cognitive complaints (SSMQ), depressive symptomatology (BDI-II, HAM-D), depressive status (RDQ), rumination (RRS-10) and quality of life (QLDS) will be assessed. Moreover, all patients will receive standard clinical care. Patients will receive standard pharmacological relapse prevention, under the responsibility of their treating physician, started during (nortriptyline) or after (lithium) ECT by combining nortriptyline (50-150 ng/ml) and lithium (≥0.5 mmol/l) for MDD and lithium (≥ 0.5 mmol/l) alone for BDD. In case of contra-indication for nortriptyline and/or lithium, alternative relapse prevention options will be explored by the treating physician. As is standard clinical care, monthly lithium dosing and testing of renal and thyroid function will be performed by the treating physician. If renal function deteriorates during follow-up this should be reported as an adverse event and may necessitate lithium discontinuation. Before dismissal from the ward participants will receive a two-session CBT-based relapse prevention group therapy and individual counseling by a social worker. Only patients who achieve a 25% or more reduction in BDI-II or HAM-D scores [36] will be randomly assigned to one of two conditions (CCT or active control), see Fig. 1 as cognitive control training has evidence as a relapse prevention strategy and not as a treatment for MDE. Non-responders to ECT will be excluded to allow further clinical treatment for MDE outside of this study protocol. After completion of the CCT, relapse of depression will be monitored for a period of 6 months using mobile assessments of depression (every 2 weeks) and telephone interviews in case of possible relapse. If there is an indication of relapse, patients will be assessed by the semi-structured Mini International Neuropsychiatric Interview (MINI v7.0.0) depressive episode module (A) and referred for psychiatric assessment to their treating psychiatrist for adequate clinical care if necessary. Patients who relapse will be excluded only if they receive a subsequent ECT-course as this might affect cognitive parameters. At three and 6 months, a more extensive evaluation will be performed investigating cognitive parameters (SSMQ), depressive symptomatology (BDI-II, HAM-D), depressive status (RDQ), rumination (RRS10) and quality of life (QLDS). We refer to Fig. 2 for an overview of the study design and Table 1 for the trial registration data set.

**Table 1:** World Health Organization Trial Registration Data Set

Data category	Information
Primary registry and trial identifying number	Clinicaltrials.gov: NCT04383509
Date of registration in primary registry	12 May 2020
Secondary identifying numbers	NA
Source(s) of monetary or material support	King Baudouin Foundation Ghent University University Hospital Ghent
Primary sponsor	Ghent University
Secondary sponsor(s)	University Hospital Ghent
Contact for public queries	Dr. Nele Van de Velde, nele.vandevelde2@uzgent.be
Contact for scientific queries	Dr. Nele Van de Velde, nele.vandevelde2@uzgent.be
Public title	RCT for Electroconvulsive Treatment Followed by Cognitive Control Training (ECT-CCT)
Scientific title	Cognitive remediation following electroconvulsive therapy in patients with treatment resistant depression: randomized controlled trail of an intervention for relapse prevention – Study Protocol
Countries of recruitment	Belgium
Health condition(s) or problem(s) studied	Depressive relapse following succesful electroconvulsive treatment (ECT) Cognitive functioning post-ECT
Intervention(s)	Active comparator:cognitive control trainingPlacebo comparator: training without focus on cognitive control
Key inclusion and exclusion criteria	• Ages eligible for study: ≥18 yearsSexes eligible for study: bothAccepts healthy volunteers: no**Inclusion criteria**: age between 18 and 70 years old, current major depressive episode with treatment resistance, eligibility and consent for ECT treatment, ability to provide consent to study **Exclusion criteria**: neurodegenerative disorder or Montreal Cognitive Assessment (MOCA) < 18, catatonia, schizophrenia, alcohol use disorder in previous year, prior ECT treatment, insufficient computer knowledge or analphabetism
Study type	InterventionalAllocation: randomizedIntervention model: parallel assignmentMasking: double blind (Participant, Outcomes Assessor)Primary purpose: treatment
Date of first enrolment	June 2020
Target sample size	88
Recruitment status	Recruiting
Primary outcome(s)	Change in severity of depressive symptoms, clinician-rated (HAM-D) Change in severity of depressive symptoms, self-reported (BDI-II) Change in depressive symptoms and quality of life, self-reported (RDQ) Time Frame:1–7 days before first ECT session, 1–7 days post ECT completion, 1–7 days after intervention/placebo completion, 3 months after ECT completion, and 6 months after ECT completion
Key secondary outcomes	Subjective memory complaints (SSMQ) Time Frame:1–7 days before first ECT session, 1–7 days post ECT completion, 1–7 days after intervention/placebo completion, 3 months after ECT completion, and 6 months after ECT completion Quality of Life in Depression Scale (QLDS) Time Frame:1–7 days before first ECT session, 1–7 days post ECT completion, 1–7 days after intervention/placebo completion, 3 months after ECT completion, and 6 months after ECT completion Rumination (RRS-10) Time Frame:1–7 days before first ECT session, 1–7 days post ECT completion, 1–7 days after intervention/placebo completion, 3 months after ECT completion, and 6 months after ECT completion Cognition (CANTAB) Time Frame:1–7 days before first ECT session, 1–7 days post ECT completion, 1–7 days after intervention/placebo completion, 3 months after ECT completion, and 6 months after ECT completion Cognition (Paced Auditory Serial Addition Task - PASAT) Time Frame:1–7 days before first ECT session, 1–7 days post ECT completion, 1–7 days after intervention/placebo completion, 3 months after ECT completion and 6 months after ECT completion Acceptability and satisfaction: Qualitative interviews Time Frame: 1–30 days after CCT training completion Time to relapse Time Frame:Up to 6 months monitoring Facial features from video footage Time Frame:2 weeks during CCT or placebo intervention

**Table 2:** Summary of protocol amendments

Protocol version	
1. December 2017	Original
2. August 2018	Qualitative interviews added
3. December 2019	Overall changes to protocol - Changes in outcome parameters (SSMQ, RDQ) - Changes to aPASAT training - Inclusion of facial videotaping - Extension of study duration

#### Intervention group

Patients in the intervention group will start the CCT training after completion of ECT with a maximum time interval of 7 days. They will perform ten sessions of the adaptive PASAT (aPASAT) on a computer in the clinical center, completing five sessions per week (400 trials per session) for a period of 2 weeks using Inquisit 5 (Millisecond, 2016). During the adaptive PASAT, participants are confronted with a continuous stream of digits (auditory; 1–9), during which they continuously need to respond to the sum of the last two heard digits. The task starts using an inter-trial interval (ITI) of 4000 ms. Every four consecutive correct responses, the ITI decreases with 100 ms, reflecting an increase in task difficulty. Vice versa, following four consecutive incorrect responses, task difficulty decreases as shown by an increase in ITI of 100 ms. Throughout the CCT task, facial video footage will be collected. Patients who complete at least 6 CCT sessions will be regarded as CCT-completers. Patients who fail to complete more than half of the CCT sessions will be excluded. To date there is no data indicating the minimum amount of completed aPASAT training sessions for optimal results.

#### Control group

Patients in the control group will start an active placebo training after completion of ECT with a maximum time interval of 7 days. The active placebo task consists of a speed of response task that shows high similarity to the experimental condition but lacks training of cognitive control. That is, participants are confronted with a continuous stream of digits (auditory; 1–18) and are instructed to immediately respond to the most recently heard digit [37]. Prior research confirmed that this condition controls for non-specific effects of the training and motivational issues [37, 38]. Patients will perform the sessions on a computer, and complete five sessions per week (400 trials per session) for a period of 2 weeks during which facial video footage will be collected.

#### Participant retention and withdrawal

Once a participant is randomized, investigators will make every reasonable effort to follow the patient for the entire 6-month follow-up period. During the cognitive control training patients will be motivated daily by researchers to complete their training. Participants have 14 days to complete 10 training sessions. The rate of loss-to-follow-up is estimated at 5 to 10% after completion of ECT treatment. Systematic methods and reminders for contacting patients, scheduling appointments, and monitoring retention will be developed by the study site.

Participants may withdraw consent for study participation for any reason at any time. For participant safety reasons, investigators may also withdraw participants from the study at any time. In case of non-adherence or non-retention, a final outcome assessment will be planned, if possible, collecting following data; SSMQ, BDI-II, HAMD, RDQ, RRS-10, QLDS and reasons for non-adherence or non-retention.

#### Adverse events

At each control visit, adverse events will be routinely screened for by asking a non-leading question to the patient such as: ‘Have you had any health problems since your last visit?’. In case of an emergency, the principal investigator will be contacted. Serious adverse events occurring during the clinical trial must be reported within 24 h to the principal investigator. If the principal investigator is not available, patients are referred to the on-call psychiatrist at the Ghent University Hospital. All participants are insured through a no-fault insurance policy conform Belgian law for human experiments. Insurance information and all contact details are provided in the informed consent document.

### Assessment

#### Depression related measures

##### HAM-D

The Dutch version of the HAM-D will be used to provide an objective measure of depressive symptoms and severity of depression. The HAM-D is a clinician rated questionnaire with 17 items scored on a three or five-point Likert-type scale. The questionnaire includes items concerning general symptoms of depression, as well as items specifically focused on mood, insomnia, and suicidal ideations [39, 40].

##### BDI-II

The Dutch version of the BDI-II will be used to measure depression severity, to monitor clinical response, remission, and possible relapse. The BDI-II is a 21-item self-report questionnaire with good validity and reliability; the internal consistency was described as around 0.9 and the retest reliability ranged from 0.73 to 0.96 [41, 42].

##### RDQ

Moreover, the Remission from Depression Questionnaire (RDQ) will inform about seven domains: symptoms of depression, non-depressive symptoms, features of positive mental health, coping ability, functioning, life satisfaction, and a general sense of well-being. The RDQ is a 41-item questionnaire showing good validity and allowing a broader perspective conform a biopsychosocial approach instead of focusing only on depressive symptoms [43, 44].

The HAM-D, BDI-II and RDQ will be completed at inclusion, after completion of the ECT course, after the intervention (active placebo or CCT) and at 3 and 6 months post-ECT (Fig. 1).

#### Rumination

##### RRS

The 10-item Dutch version of the Ruminative Response Scale (RRS-10) will be used to monitor rumination or repetitive negative thinking as this is a well-established cognitive risk factor for MDE. This self-report scale has demonstrated to be a reliable and valid instrument [45].

#### Quality of life

##### QLDS

To assess impact of ECT and cognitive control training on the perceived quality of life and emotional wellbeing, the Dutch version of the QLDS [46, 47] will be used. This 34-item questionnaire, developed by qualitative interviews, asks patients to rate statements regarding fulfillment of human needs such as ‘I take good care of myself’ and ‘I like to know what is going on in the world’. The QLDS, which has demonstrated good reliability and internal consistency, will be completed at inclusion, after completion of the ECT course, after the intervention (active placebo or CCT), and at 3 and 6 months post-ECT (Fig. 1).

#### Cognitive functioning

##### CANTAB

In order to assess the patients’ cognitive functioning, three tests of the Cambridge Neuropsychological Test Automated Battery (CANTAB®) were selected to cover (working) memory and executive functioning: Pattern Recognition Memory (measuring visual pattern recognition memory) and One Touch Stockings of Cambridge (measuring spatial spanning and working memory). A third test, Motor Screening was included to measure general sensorimotor skills. CANTAB® tests will be measured before ECT, after ECT, and after CCT completion (Fig.1).

##### PASAT

A non-adaptive version of the PASAT task will be used to assess task-specific cognitive transfer following (as compared to before) CCT [25, 48]. In line with the adaptive PASAT, patients are confronted with a continuous stream of digits and have to respond to the last two heard digits. Following a practice phase consisting of 10 trials, participants will complete a test phase which consists of three blocks of 60 trials each. Task difficulty increases over the different blocks, using an ITI of 4000, 2000, and 1500 ms for the first, second, and third block respectively.

##### SSMQ

To assess subjective cognitive functioning the Dutch version of the Squire Subjective Memory Questionnaire (SSMQ) will be administered at inclusion, after completion of the ECT course, after the intervention (active placebo or CCT) and at 3 and 6 months post-ECT (Fig.1). This 18-item self-report questionnaire uses a Likert scale to assess various aspects of subjective memory. The SSMQ is the most widely used scale for measuring subjective memory post-ECT [49].

##### Qualitative interviews

Qualitative interviews will be conducted to assess subjective memory complaints, acceptability of the cognitive remediation task and satisfaction of patients and family members (e.g., spouse). Interviews will be conducted after completion of CCT or placebo training.

#### Assessment of depression relapse

Patients will receive a text message once every 2 weeks (except at 3 and 6 months post-ECT; when they are invited for a more thorough interview at the hospital) to enquire whether they experienced either core symptom of depression over the past 2 weeks: 1) Have you been consistently depressed or down, most of the day, nearly every day, for the past 2 weeks?; 2) In the past 2 weeks, have you been much less interested in most things or much less able to enjoy the things you used to enjoy most of the time? . Answer can be yes or no. The text messages will be sent using SurveySignal at 18.00 h containing a link to a two question LimeSurvey questionnaire. If the patient does not respond within the first hour, a reminder text message will be sent. If the patient either does not respond to the screening questions within a 24-h timeframe, or answers either of the questions affirmatively, the patient will be called by the researcher to conduct the semi-structured Mini International Neuropsychiatric Interview (MINI v7.0.0) depressive episode module (A). If a relapse has concluded, patients will be referred for psychiatric assessment to their treating psychiatrist for adequate clinical care. These phone calls will be recorded and stored for later evaluative purposes and diagnosis by an independent rater.

### Sample size calculation

Using G*Power version 3.1.9.2, we estimated the sample size necessary to detect a moderate effect of CCT following ECT on our primary outcome measures relapse of depression and severity of depressive symptomatology. That is, initial findings suggest a moderate effect of CCT on risk for recurrence of depression in a sample of remitted depressed patients (Odds Ratio = 0.38; Hoorelbeke, Van den Bergh, Wichers, De Raedt, & Koster, under review). A sample size of *N* = 88 would be required to detect a moderate effect (*w* = 0.3) using a Pearson Chi-Square test, with α = .05 and 1 – β = .80. Sensitivity analysis suggests that the required *N* = 88 for analysis of recurrence of depression, would allow to detect an effect-size of *f* = .125, ranging from low to moderate, for the indicators of severity of depressive symptomatology using a 4 (time) × 2 (condition) repeated measures ANOVA with a correlation between the repeated measurements of *r* = .50, α = .05 and 1 – β = .80. As such, we aim to recruit participants until *N* = 88 (partial) responders have completed the experimental manipulation following ECT.

### Data management and analyses

Participant files, including informed consent forms, will be stored in a locked cabinet with restricted access for a period of 20 years after study completion.

We will use a 4 (time: T2, T3, T4, T5) by 2 (condition: active placebo CCT; real CTT) repeated measures design. The significance level will be set at α = .05.

For our main analyses, we will rely on intention-to-treat analysis (ITT) to model effects of CCT on the outcome measures of interest, using the Last-Observation-Carried-Forward (LOCF) method to handle missing data (due to non-response, drop-out or relapse of depression). Where adequate, effects of CCT on our cognitive measures and questionnaire data will be modeled using repeated measures Analysis of Variance (ANOVA) or Covariance (ANCOVA). Significant interaction effects will be further explored using independent and paired samples *t*-tests. Effects of CCT on relapse of depression will be modeled using a Pearson Chi-Square test. In addition, we will conduct survival analyses (Kaplan Meier estimator) to model time to relapse, using the log-rank test to compare the obtained survival curves.

Qualitative data analyses methods will be used to analyze the interviews regarding satisfaction, acceptability, and appropriateness of the design.

Exploratory analyses will be used to investigate the facial video footage data collected during the ten sessions of CCT or active placebo. From the footage, we will deduce facial landmarks and reactions during the performance of the training as an indication of frustration, engagement and general mood, whereas different factors could be of influence on the effectivity of the training. More specifically, we expect to see differences in, e.g., general facial valence, facial muscle tension and frustration.

## Discussion

The focus of this study is to investigate cognitive control training as a novel relapse prevention strategy after response to ECT treatment and increasing acceptability of ECT by reducing cognitive side-effects. Cognitive impairment is an important symptom cluster in affective disorders and may continue to affect functional outcomes in a negative matter even after achieving remission of mood symptoms [9]. Cognitive side-effects after ECT are reported by patients as the most disturbing due to their negative impact on quality of life [11]. To date there is only one RCT targeting cognitive-side-effects after ECT by training specific cognitive functions. Choi et al. (2017) conducted a double-blind study investigating the efficacy of a specific memory training targeting anterograde and retrograde memory reporting negative findings [50]. They attributed their null findings in part due to the progression of ECT techniques, such as ultra-brief pulse width and right unilateral (RUL) electrode placement, with a more favorable side-effect profile regarding cognition. In our study patients will receive RUL ECT as this is standard clinical practice at our service. Electrode placement might be switched to bitemporal (BT) stimulation depending on the clinician’s decision if there is no response to treatment. Our sample will thus contain both patients who received RUL and BT ECT. We expect patients treated with BT ECT to experience more cognitive side-effects compared to RUL-ECT treated patients. Even more important, CCT training has a different target, training cognitive control or working memory versus recovering episodic memories and memory skills training. Training cognitive control has shown robust results in patients with depressive episodes as an add-on preventive and/or curative intervention in the treatment of depression.

High relapse rates continue to be a clinical challenge in patients with major depressive episodes and more specifically in those patients treated with ECT due to the treatment resistant and severe nature of their condition. There is an urgent need for novel relapse prevention strategies. Cognitive control training has demonstrated promising results in diminishing relapse rates in patients with depressive symptoms. Reducing relapse and improving acceptability of ECT through reduction of cognitive side effects would be highly desirable outcomes.

## Data Availability

All investigators will have access to the password protected data sets. The datasets generated and/or analyzed during the current study are not publicly available due to confidentiality issues but are available from the corresponding author upon reasonable request.

## Abbreviations

aPASAT: Adaptive Paced Auditory Serial Addition Task
BDD: Bipolar depression
BDI-II: Beck depression Inventory, 2nd edition
BT: Bitemporal
CANTAB®: Cambridge Neuropsychological Test Automated Battery
CCT: Cognitive control training
c-ECT: Continuation electroconvulsive therapy
DSM-5: Diagnostic and Statistical Manual
ECT: Electroconvulsive therapy
HAM-D: Hamilton Depression Rating Scale
MDD: Major depressive disorder
MDE: Major depressive episode
MINI: Mini International Neuropsychiatric Interview
RCT: Randomized controlled trial
MMSE: Mini mental state examination
MOCA: Montreal Cognitive Assessment
RDQ: Remission from Depression Questionnaire
RUL: Right unilateral
RRS-10: Rumination Response Scale 10-items
SSMQ: Squire subjective memory questionnaire
QLDS: Quality of life in Depression Scale
